# Detecting the Subtle Photo-Responsive Conformational Bistability of Monomeric Azobenzene Functionalized Keggin Polyoxometalates by Using Ion-Mobility Mass Spectrometry

**DOI:** 10.3390/molecules27123927

**Published:** 2022-06-19

**Authors:** Bo Qi, Luran Jiang, Sai An, Wei Chen, Yu-Fei Song

**Affiliations:** State Key Laboratory of Chemical Resource Engineering, Beijing University of Chemical Technology, Beijing 100029, China; bqi@mail.buct.edu.cn (B.Q.); lr_jiang2021@163.com (L.J.); ansai@mail.buct.edu.cn (S.A.)

**Keywords:** polyoxometalates, azobenzene, photo-responsive, ion-mobility mass spectrometry, shape characterization

## Abstract

Accurately characterizing the conformational variation of novel molecular assemblies is important but often ignored due to limited characterization methods. Herein, we reported the use of ion-mobility mass spectrometry (IMS/MS) to investigate the conformational changes of four azobenzene covalently functionalized Keggin hybrids (azo-Keggins, compounds **1**–**4**). The as-prepared azo-Keggins showed the general molecular formula of [C_16_H_36_N]_4_[SiW_11_O_40_(Si(CH_2_)_3_NH–CO(CH_2_)_n_O–C_6_H_4_N=NC_6_H_4_–R)_2_] (R = H, *n* = 0 (**1**); R = NO_2_, *n* = 0 (**2**); R = H, *n* = 5 (**3**); R = H, *n* = 10 (**4**)). The resultant azo-Keggins maintained stable monomeric states in the gas phase with intact molecular structures. Furthermore, the subtle photo-responsive trans-cis conformational variations of azo-Keggins were clearly revealed by the molecular shape-related collision cross-section value difference ranging from 2.44 Å^2^ to 6.91 Å^2^. The longer the alkyl chains linkers were, the larger the conformational variation was. Moreover, for compounds **1** and **2**, higher stability in *trans*-conformation can be observed, while for compounds **3** and **4**, bistability can be achieved for both of them.

## 1. Introduction

Azobenzene (Azo) and its derivatives have been widely investigated as promising photochemical systems, owing to their properties of reversible *trans-cis* isomerization upon light irradiation, which further results in reversibly changed physical and optical properties such as the solubility, dipole moment, surface free energy, and mechanical actuation behaviors [1,2,3,4,5,6]. By attaching azo groups, photochemical properties could be introduced to new functional materials [7]. In recent years, polyoxometalates (POMs), a class of anionic molecular metal oxide clusters with intriguing physical properties and nanometer size [8,9,10,11,12,13,14], have been applied to combine with azo groups. The resultant azo-based polyoxometalates (azo-POMs) have shown fascinating photo-responsive behaviors and have been applied for to a broad area, such as lyotropic or thermotropic liquid crystal, supramolecular self-assembly, and catalysis [15,16,17,18,19]. Different from small molecular azo, macromolecular azo-POMs show relatively complicated physical properties because of the introduction of more parameters such as large molecular size, multiple charges, steric hindrance effect, electronic effect, etc. [20]. Although the reversible *trans-cis* conformational change of molecules was studied, the detection means were still limited to UV-Vis or NMR spectroscopy [19,21]. Moreover, the shape’s variation was inevitably involved during conformational changes; however, this factor is often ignored. Therefore, it is important to develop novel characterization methods for obtaining more structural information about these azo-POM macromolecules.

The technique of ion mobility mass spectrometry (IMS/MS) has been demonstrated to be useful for larger and more complex biological molecules as early as the 1990s [22,23,24,25]. With the development of ion mobility separation technology and high-resolution electrospray ionization mass spectrometry systems, the applications of IMS/MS were expanded to the investigation of polymers, proteomics, protein digest mixture, and carbon clusters as well as the supramolecular assembly of POMs [26,27,28,29,30,31,32]. By coupling MS instruments that separate ions on the basis of mass-to-charge ratio and IMS instruments that separate ions based on size-to-charge ratio, a two-dimensional mobility-mass spectrum could be obtained with information on size, charge, shape, and mass dimensions. In particular, the isomers or conformers have different shapes while very similar mass information can be separated in the mobility space [18,29]. During the past five years, the conformational variation of azobenzene was studied by using IMS/MS. However, most related studies were focused on the organic azo-based small molecules with CCS values less than 277 Å^2^ [33,34]. In contrast, few works were reported for investigating the azo-based large macromolecules, which may bring a more complex while fascinating isomerization behavior. Therefore, it should be interesting to study azo-based POMs by using IMS/MS, due to their 1–10 nm molecular sizes, multiple negative charges, and redox properties, which were quite different with small azo molecules.

To best of our knowledge, the only work for studying the azo-POMs by IMS/MS was reported by Song, Cronin, and co-workers [18]. Taking the advantage of IMS/MS, the high CCS value of 600 Å^2^ and the bistability conformational variations of azo-modified Mn-Anderson POMs (denoted as azo-Andersons) were observed. A significant difference in the collision cross-section (CCS) value of the oligomeric compounds was observed between the *trans*- and *cis*-conformation of the azo groups. However, these oligomeric structures involved the influence of intermolecular arrangement, which may overestimate shape differences caused by the isomerization of the single molecule itself. Therefore, applying the novel azo-POM systems with relatively stable monomeric molecules is crucial for detecting the bistability nature of azo-based macromolecules with the more subtle and intriguing reversible variation. Moreover, it was also intriguing to develop the substituents effect on the stability of *trans-* and *cis-*isomers.

## 2. Results and Discussion

**Figure 1:**
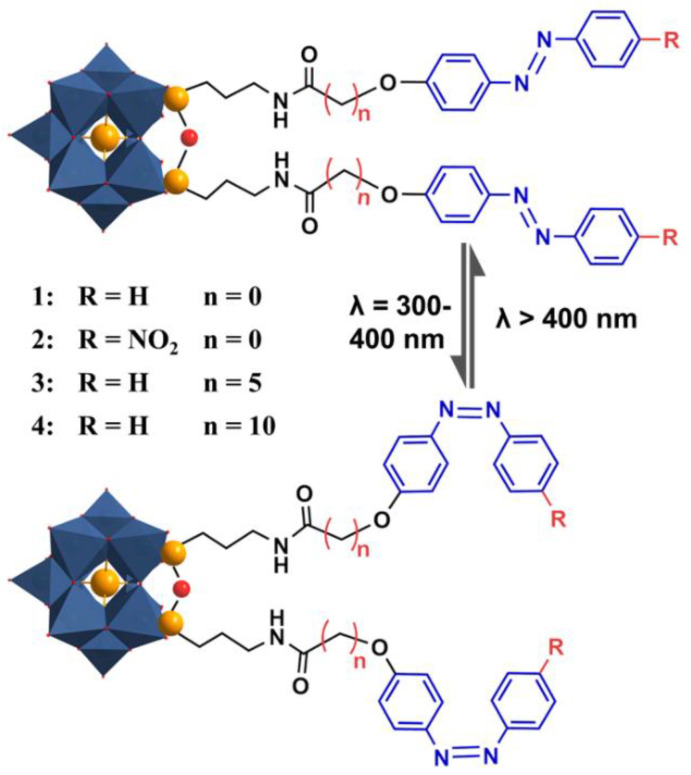
The lacunary Keggin cluster [SiW_11_O_39_]^8−^ functionalized by photo-responsive azobenzene groups with different lengths of linkers and substituent groups (Compound **14**). Upon UV irradiation, the azo bond switches from *trans*- to *cis*-conformation. Under visible light irradiation, the bond switches back again.

In this work, four azo ligands with different lengths of alkyl linkers and substituent groups were covalently grafted onto the lacunary Keggin of [SiW_11_O_39_]^8−^ (SiW_11_) (denoted as azo-Keggins). The as-prepared azo-Keggins showed the general molecular formula of [C_16_H_36_N]_4_[SiW_11_O_40_(Si(CH_2_)_3_–NH–CO(CH_2_)_n_O–C_6_H_4_N=NC_6_H_4_–R)_2_] (R = H, *n* = 0 (**1**); R = NO_2_, *n* = 0 (**2**); R = H, *n* = 5 (**3**); R = H, *n* = 10 (**4**)). Different from the previously reported azo-Andersons, in which two azo ligands were attached to the opposite sides of MnMo_6_O_18_, the resultant azo-Keggins contained two azo ligands on the same sides of SiW_11_ (Figure 1). Considering strong intermolecular interactions was the prerequisite for the formation of large aggregates in the solution or gas phase; these azo-Keggins were expected to have weak intermolecular interactions due to hindrance from Keggin clusters with large molecular size and strong electrostatic repulsion.

**Figure 2:**
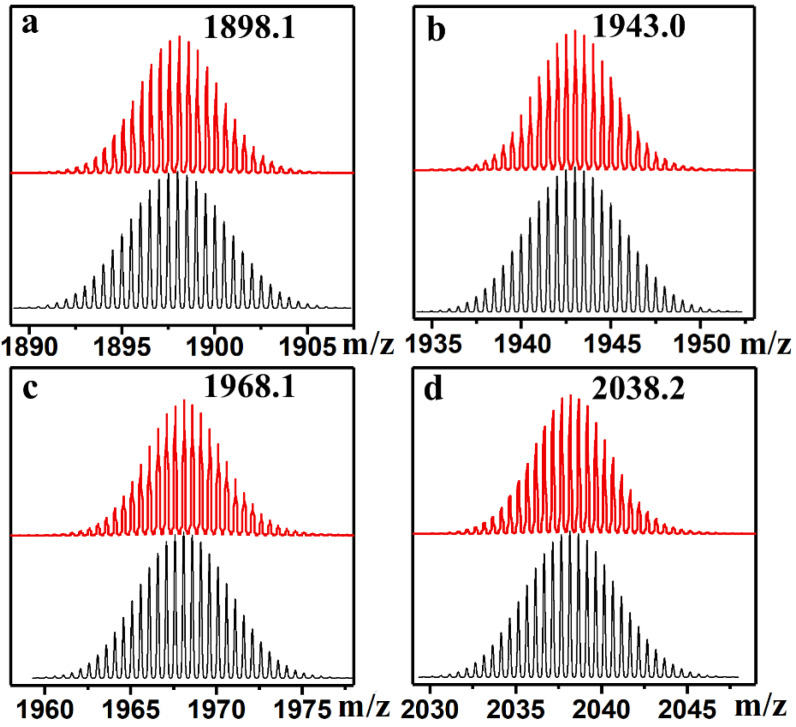
ESI-MS spectra of the compound **1**–**4** (red peaks in **a**–**d**) showing the *m/z* towards fragment ions of [**X**_**1**–**4**_ + 2TBA]^2−^ (**X_1–4_** = anionic part of **1–4**). The corresponding simulated isotopic patterns of the [**X_1–4_** +2TBA]^2−^ were indicated as the grey peaks in **a**–**d**.

The azo-POMs can be detected by electrospray ionization mass spectrometry (ESI-MS), as the aggregates commonly yielded multiple species with similar *m*/*z* resulting in overlapping envelopes in the mass spectra [18]. The ESI-MS spectra of compounds **1**–**4** were acquired by directly using their acetonitrile solutions. In each case, a series of notable peaks was observed and can be well assigned to the corresponding fragment ions, demonstrating that these hybrid structures were successfully prepared and remained intact both in the solution and gas phase. As shown in Figure 2, compounds **1**–**4** showed MS signals at *m*/*z* 1898.1, 1943.0, 1968.1, and 2038.2, which can be assigned to the fragment ions of [**X**_**1**–**4**_+2TBA]^2-^ (**X**_**1**–**4**_ = the anionic part of compounds **1**–**4**; TBA = tetrabutylammonium). All four peaks provided the unambiguous isotopic distribution envelopes without any similar overlapping fashion, suggesting the monomeric state of azo-Keggins. Full spectra and a list of identified peaks are provided in the Figures S1–S4.

**Figure 3:**
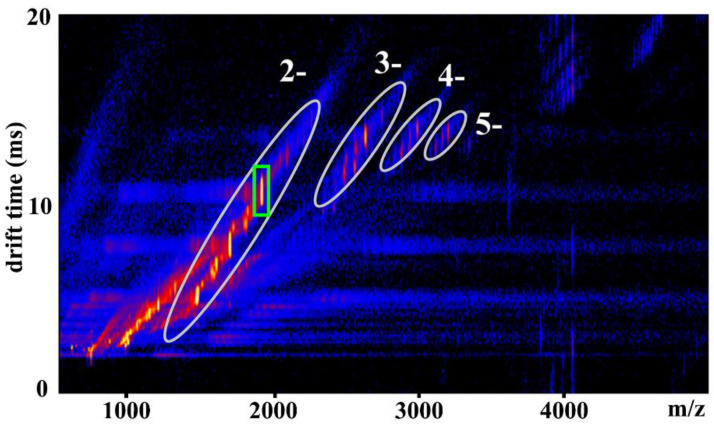
The 2D IMS/MS spectrum of compound **1**. The diagonal lines of similarly charged species were encircled by ellipsoids and the charges of these species were given in white. The peak in green rectangle was assigned to [**X_1_** + 2TBA]^2−^.

After confirming the monomeric state of azo-Keggins with intact molecular structure, IMS/MS measurements were applied to analyze their bistability conformation and reversible dynamics, considering that MS was unable to provide the information on shape. A commercially available travelling-wave ion-mobility spectrometer (TWIMS) was coupled with mass measurements to separate the ions according to their mobility and to derive the collision cross-section (CCS) data of the ions. Figure 3 showed the 2D IMS/MS spectrum of compound **1**, with the peak intensity displayed by a color-coded logarithmic scale. The *x*-axis represented the *m*/*z* range from MS, and the *y*-axis represented the drift time from IMS. As we might expect, the spectrum of compound **1** showed a clearer situation than that of azo-Andersons, where larger oligomeric structures or the higher charge states were minor. Same results were observed in the 2D IMS/MS spectra of **2**–**4** both in their *trans*- and *cis*-conformations (Figures S5–S12). The intense (yellow) line of the peaks could be easily assigned to the individual cluster ions as observed in ESI-MS. All these peak envelopes could be assigned. The intensive peak at *m/z* = 1989.1 and drift time = 10.8 ms was assigned to the monomeric Keggin hybrids: [**X_1_** + 2TBA]^2−^ (**X_1_** = anionic part of **1**). The corresponding higher charge peaks were assigned to minor aggregates: [**2X_1_** + 5TBA]^3−^, [**3X_1_**+8TBA]^4−^, and [**4X_1_**+11TBA]^5−^ (Figure 3).

**Figure 4:**
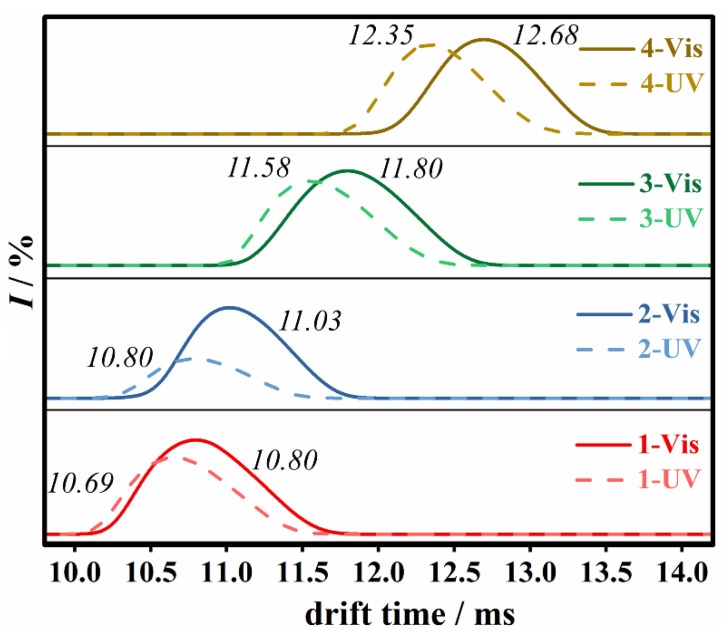
Comparison of the drift time graphs of the main MS peaks of compounds **1**–**4** with visible (solid lines) and UV (dash lines) irradiation. The spectra were consistent for all three repeated cycles.

With the further inspection of the IMS/MS spectrum of compound **1**, the conformational variation of these azo functionalized POM macromolecules could be observed. It was well known that the conformation of the azo bond can change from *trans* to *cis* upon UV irradiation at 365 nm and reversibly recovered back to *trans* conformation when the sample was exposed to visible light. The two conformations were relatively stable under the constant light condition, which enabled the detection of the bistability of azo-Keggins and the reversible transformation process. Additional UV-Vis and ^1^H NMR experiments were conducted before and after UV irradiation at 365 nm. Moreover, the kinetic curve of the UV absorbance with time increasing was provided to follow the isomerization process (Figure S20). The results showed that almost all *trans*-isomers were changed to *cis*-isomers after 5 h UV light irradiation. The *cis*-isomer samples were immediately measured by IMS/MS under the exclusion of light. Figure 4 showed the drift time spectra for the main MS peaks of anionic part of compounds **1**–**4** with two TBA cations [**X_1–4_** + 2TBA]^2−^. Before and after UV irradiation, the drift time peaks (as indicated by green rectangle in Figure 3) manifested a clear shift, suggesting that the shape of compounds was changed. In IMS, the drift velocity of the ions was proportional to the electric field, and proportionality constant *K* was related to the CCS of ions [23]. On the basis of the CCS value that can be measured directly from the drift time, information about the chemical structure and 3D conformation of the ions was further provided (Tables S1–S4 Supplementary Materials).

Furthermore, the stability of specific isomers can be reflected in the intensity of drift time peaks. As shown in Figure 4, **1-UV** showed a lower peak intensity than its isomer **1-Vis**, suggesting the lower stability of *cis* conformation than that of *trans* conformation. The decreased stability was attributed to the linkage of the Keggin POM cluster, which was considered a strong electron-withdrawing group [35]. Comparing the peak intensity of **2-UV** and **2-Vis**, the terminal substituent of electron-withdrawing NO_2_ groups further decreased the stability of *cis* conformation [36]. Similar peak intensities were observed between two conformational isomers toward **3** and **4**, suggesting that the effect of substituent on the stability of isomers was diminishing with the increased length of alkyl chains between POMs and azo groups. Therefore, the conformation bistability of compounds **3** and **4** could be achieved.

**Figure 5:**
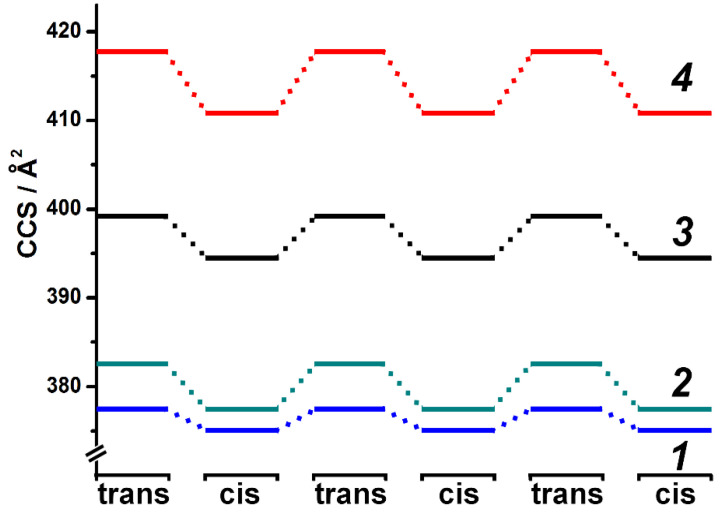
The changes in collision cross-section of compounds **1**–**4** when switching between *trans* and *cis* conformations of the azo bond. The formula of the azo-Keggin monomers was [**X_1–4_** + 2TBA]^2−^.

As shown in Figure 5, the CCS value between the *trans* and *cis* conformations of compounds **1**–**4** showed the difference ranging from 6.91 Å^2^ for the linker with the longest alkyl chain to 2.44 Å^2^ for the smallest compound. Compared with the dimeric Anderson azo-POMs (CCS difference from 26 Å^2^ to 13 Å^2^), monomeric azo-Keggins exhibited a higher resolution difference, which could more precisely reflect the subtle conformational change of macromolecules derived from the azo group itself. The CCS differences for compounds **1**–**2** were 2.44 Å^2^ and 5.07 Å^2^, respectively, which were consistent with both of the roughly calculated conformational difference of the azo or nitro-azo groups by ChemBio3D Software and 2.1 Å^2^ CCS difference of the conformational variation of the protonated azo reported in the literature [35]. As for compounds **3**–**4**, the greater CCS difference value was attributed to the more significant shape variation caused by flexible alkane chains. Therefore, for macromolecular azo-Keggins, the conformational change originated mainly from the transformation of organic azo ligands, while the influence from bulky POM clusters was minor. The IMS/MS measurement toward compounds **1**–**4** with reversible UV/Vis irradiation was repeated three times, and the resulting spectra were consistent through all repetitions of the isomerization, suggesting reproducibility.

It is worthwhile to note that our previous work reported the investigation of the IMSMS study of azo-Anderson assemblies [18]. In contrast, the azo-Keggin assemblies reported herein showed very different results: (1) The resultant azo-Keggins could stabilize the monomeric state, while azo-Anderson favored to form aggregates (dimer, trimer, and tetramer, etc.). Moreover, the conformational variation of azo-Keggins was more precisely reflected in IMS/MS results with a smaller CCS difference (2.44 Å^2^ to 6.91 Å^2^) than that of azo-Anderson aggregates with a large CCS difference (13 Å^2^ to 26 Å^2^), which was due to the influence of the intermolecular arrangement. (2) The azo-Andersons with substituents of electron donor groups (such as alkoxyl chains) showed a higher stability of *cis-*conformation compared with that of a *trans-*conformation. As complementary, azo-Keggins with a substituent of electron-withdrawing groups exhibited a higher stability of *trans-*conformation than that of *cis-*conformation. When long alkyl chains were present in azo-Keggin assemblies (-C5 and -C10 alkyl chains were linked between azo and POMs), the bistability of both *trans-* and *cis-* isomers can be achieved.

## 3. Materials and Methods

### 3.1. General Materials

All materials and reagents were purchased from commercial sources and used without further purification. K_8_SiW_11_O_39_·13H_2_O and azo-functionalized compounds **1**, **3** and **4** were prepared according to the literature [15]. Fourier transform infrared (FT-IR) spectra were recorded on JASCO FT-IR 410 spectrometeror or a JASCO FT-IR 4100 spectrometer (JASCO, Tokyo, Japan). ^1^H NMR spectroscopy was performed on a Bruker DPX 400 spectrometer (Bruker, Zurich, Switzerland) using the solvent’s signal as an internal standard. Quantitative elemental analyses of C, H, and N were determined by microanalysis services within the College of Chemistry, Beijing University of Chemical Technology.

### 3.2. IMS/MS Studies and Determination of Collision Cross-Sections

All measurements were performed on a Synapt™ G2 HDMS™ from Waters (Massachusetts, USA) with equipment of a Quadrupole and Time-of-flight (Q/ToF) module for MS analysis. The IMS section is a travelling-wave IMS, which is located between the Q- and ToF-sections consisting of a trap cell, an ion-mobility cell, and a transfer cell. All compounds were directly dissolved in HPLC-grade acetonitrile at a concentration of 10^−5^ M. The solutions were filtered through a syringe filter (0.2 µm) before being injected into the spectrometer via a Harvard syringe pump at a flow rate of 5 µL·min^−1^. The parameters for IMS/MS measurement of all compounds have been set up with the following: ESI capillary voltage: 1.93 kV; sample cone voltage: 10 V; extraction cone voltage: 4.6 V; source temperature: 80 °C; desolvation temperature: 120 °C; cone gas flow: 15 L·h^−1^ (N_2_); desolvation gas flow: 500 L·h^−1^ (N_2_); source gas flow: 0 mL·min^−1^; trap gas flow: 2.5 mL·min^−1^; helium cell gas flow: 200 mL·min^−1^; IMS gas flow: 90.00 mL·min^−1^; IMS DC entrance: 25.0; helium cell DC: 35.0; helium exit: −5; IMS bias: 30; IMS DC exit: 0; IMS wave velocity: 700 m·s^−1^; IMS wave height: 40 V.

Collision cross-sections (CCSs) were estimated following calibration with Equine Cytochrome C and T10 olgiothymidine to determine instrument-dependent parameters A and B from published CCSs data [5], as previously described in the literature [24]. The analysis of MS spectra was carried out using Mass Lynx V4.1 Software supplied by Waters. Driftscope V2.1 was used for the analysis of IMS/MS spectra and calibration of the drift cell for the determination of CCSs. IMS experiments were performed in -ve mode. All drift times and cross-sections are quoted for the intact 2- fragment (i.e., [**X_1_**_–**4**_ + 2TBA]^2−^) for the following reasons: (1) [**X_1–4_** + 2TBA]^2−^ forms were the largest molecular fragment in ESI-MS spectra. (2) [**X_1–4_** + 2TBA]^2−^ forms had relatively intact molecular structure instead of partial structural fragments. (3) As the characteristic peak, [**X_1–4_** + 2TBA]^2−^ existed in all IMS/MS spectra of compounds **1**–**4**.

### 3.3. Synthesis of (Bu_4_N)_4_{SiW_11_O_39_[O(SiCH_2_CH_2_CH_2_NHCOR)_2_]} (R = -OC_6_H_4_NNC_6_H_5_NO_2_, Compound **2**)

When (*E*)-4-((4-nitrophenyl)diazenyl)phenyl (3-(triethoxysilyl)propyl)carbamate (0.77 g, 1.57 mmol) was dissolved in a solution of H_2_O/CH_3_CN (*v*/*v* = 20/60 mL) at room temperature, a turbid solution was formed to which K_8_SiW_11_O_39_·13H_2_O (1.95 g, 0.60 mmol) was added. The pH of the resulting reaction mixture was slowly adjusted to 0.7 with 1M HCl, and the clear solution was stirred overnight. Then, CH_3_CN was evaporated and NBu_4_Br (4.20 g, 13 mmol) was added to the aqueous solution to precipitate the expected product. The precipitate was collected and washed with deionized water (100 mL), ethanol (100 mL), and diethyl ether (100 mL) before being dried in a vacuum. Yield: 2.25 g (85.8%). IR (KBr, cm^−1^): ν = 3418 (m), 2961 (m), 2873 (m), 1742 (m), 1525 (m), 1485 (m), 1344 (m), 1205 (m), 1141 (w), 1044 (m), 964 (s), 947 (s), 904 (vs.), 856 (m), 804 (s), 535 (w). ^1^H NMR (CD_3_CN-*d*_3_, 400 MHz, ppm) δ = 8.32 (d, ArH, 4H), 7.97 (d, ArH, 4H), 7.97 (d, ArH, 4H), 7.92 (d, ArH, 4H), 7.31 (d, ArH, 4H), 3.29 (t, CH_2_, 4H), 3.12 (t, CH_2_, 32H), 1.86 (s, CH_2_, 4H), 1.62 (q, CH_2_, 32H), 1.37 (m, CH_2_, 32H), 0.97 (t, CH_3_, 48H), 0.76 (t, CH_2_, 4H). ^13^C NMR (CD_3_CN-*d_3_*, 100 MHz, ppm) δ = 156.6, 155.8, 155.0, 150.2, 149.7, 125.8, 125.3, 124.2, 123.7, 59.2, 44.1, 24.3, 24.0, 20.3, 13.9, 10.8. ESI-MS (negative mode, CH_3_CN): *m*/*z*: 1943 [**X_2_** + 2TBA]^2−^. Anal. Calcd. for C_96_H_174_N_12_O_48_Si_3_W_11_ (4370.96): C, 26.38; H, 4.01; N, 3.85; Found: C, 25.91; H, 4.02; N, 3.61.

## 4. Conclusions

In conclusion, four azo-Keggins macromolecules with hybrid structures in which the photo-responsive azo moieties with different lengths of alkyl linkers and substituents were grafted onto the SiW_11_ clusters were applied to investigate their photo-responsive conformational variation properties. ESI-MS of compounds **1**–**4** showed that all fragment ions can be well assigned without observing any similar overlapping fashion in each mass envelopes, suggesting that the monomeric state of these azo-Keggins was intact both in the solution and in the gas phase. The 2D IMS/MS spectra further indicated the high stability of the monomeric state which presented as the main peak envelops. Moreover, with the reversible UV and Vis photoirradiation, the subtle conformational change of these azo-Keggins resulted in different CCS values with a higher resolution of 2.44 Å^2^. Compared with the previously reported data for large assemblies of azo-Andersons, the conformational variation results of monomeric azo-Keggins could more precisely indicate the effect of each group on the shape and stability of isomers: (1) The longer the linker’s length was, the greater the conformational variation. (2) Conformational bistability was achieved for compounds **3** and **4**, while for compounds 1 and 2 with electron-withdrawing substituents, higher stability in the *trans* conformation was observed. This work opened up a crucial new characterization dimension for which information on size, shape, charge, mass, and variation can be revealed. Providing deep insight into the shape changes of POMs into areas of self-assembly, catalysis, and responsive behavior from external stimulation such as photo, thermal, magnetic, etc., was helpful.

## Data Availability

Not applicable.

## Supplementary Materials

The following supporting information can be downloaded at: https://www.mdpi.com/article/10.3390/molecules27123927/s1, Figures S1–S4: ESI-MS spectrum of compound **1**–**4** and notable peaks assignment. Figures S5, S7, S9, and S11: 2D IMS/MS spectrum of compound **1**–**4** before UV-irradiation. Figures S6, S8, S10 and S12: 2D IMS/MS spectrum of compounds **1**–**4** after UV-irradiation. Figures S13–S16: Drift time of compounds [**X_1_**_–**4**_ + 2TBA]^2−^ before and after UV-irradiation. Tables S1–S4: Change in drift time and CCS of monomeric compounds [**X_1_**_–**4**_ + 2TBA]^2−^ when switching between the *trans* and the *cis* conformation of the azo bond. Synthesis method of organic compounds used for the synthesis of compounds **1** and **2**. Figures S17–S19: FT-IR, ^1^H NMR, and ^13^C NMR spectra of compounds **1** and **2**. Figure S20: (a) ^1^H NMR spectra of compound **2** in *d*_3_-CH_3_CN before (bottom) and after (top) UV irradiation at 365 nm. (b) UV/Vis spectra of compound **2** in CH_3_CN. (c) The kinetic curve of UV absorbance with increasing time. (d) UV absorbance at 343 nm upon alternating UV irradiation at 365 nm and visible light for five cycles. Figure S21: The models of the azo and nitro-azo groups with *trans-* and *cis-*conformations. The models were constructed by ChemBio3D Software, and the shape difference was roughly calculated by atom distance.

## Sample Availability

Samples of the compounds are available from the authors.
