# Supplementary material for: Detecting the Subtle Photo-Responsive Conformational Bistability of Monomeric Azobenzene Functionalized Keggin Polyoxometalates by Using Ion-Mobility Mass Spectrometry
Source: Molecules. 2022 Jun 19;27(12):3927. doi: 10.3390/molecules27123927 (PMC9228792; doi:10.3390/molecules27123927)
Supplement: Supplementary file 1 [file molecules-27-03927-s001.zip › molecules-1764534-supplementary.pdf]

Detecting the Subtle Photo-Responsive Conformational Bistability of Monomeric Azobenzene Functionalized Keggin Polyoxometalates by Using Ion-Mobility Mass Spectrometry

Bo Qi [1], Luran Jiang [1], Sai An [1], Wei Chen [1,*] and Yu-Fei Song [1,*]

[1] State Key Laboratory of Chemical Resource Engineering, Beijing University of Chemical Technology, 100029 Beijing, P.R. China; bqi@mail.buct.edu.cn (B.Q.); lr_jiang2021@163.com (L.J.); ansai@mail.buct.edu.cn (S.A.).

* Correspondence: chenw@mail.buct.edu.cn (W.C.); songyf@mail.buct.edu.cn (Y.F.S.)

Table of Contents

1. MS summary 2

2. 2D IMS/MS summary 4

3. Drift times and collision cross-sections before and after UV-irradiation 7

4. Characterization of compounds 1 and 2 10

5. Photo-responsive conformational variation behaviors of compound 2 13

1. MS summary

Compound **1**: $(TBA)_4[SiW_{11}O_{39}\{O(Si(CH_2)_3NHCOOC_6H_4NNC_6H_5)_2\}]$

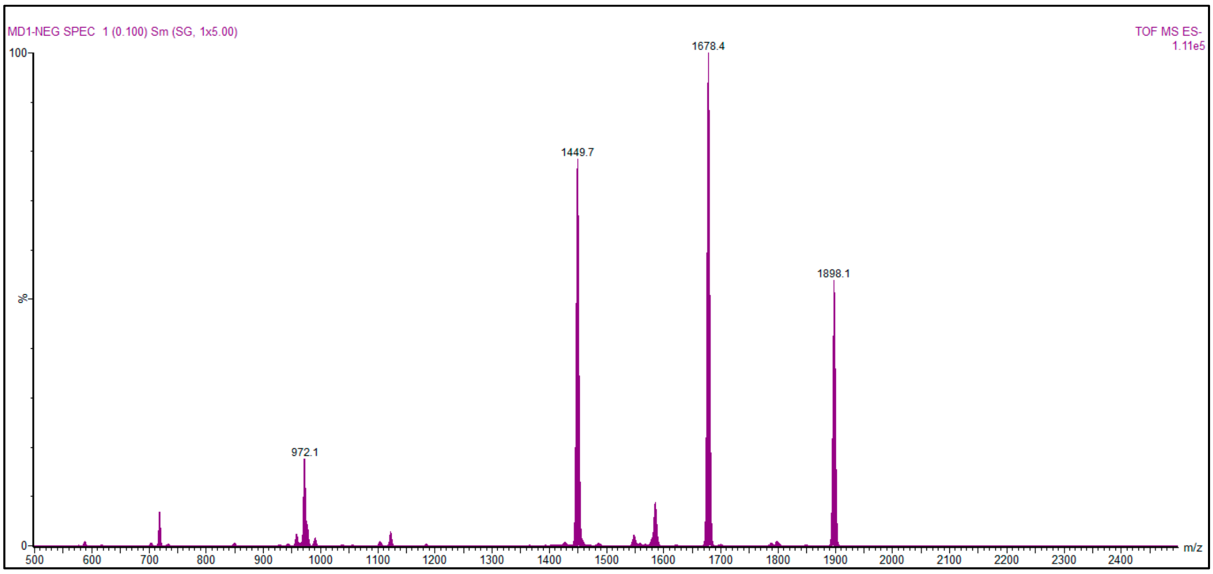

Figure S1. ESI-MS spectrum of compound **1**.

Notable Peaks:*

m/z	*z*	*Assignment*
972.1	3-	$\{H[SiW_{11}O_{39}\{O(Si(CH_2)_3NHCO)_2\}]\}$
1449.7	2-	$\{KH[SiW_{11}O_{39}\{O(Si(CH_2)_3NH_2)_2\}]\}$
1678.4	2-	$\{Na_2[SiW_{11}O_{39}\{O(Si(CH_2)_3NHCOOC_6H_4NNC_6H_5)_2\}]\}$
1898.0	2-	$\{(TBA)_2[SiW_{11}O_{39}\{O(Si(CH_2)_3NHCOOC_6H_4NNC_6H_5)_2\}]\}$

Compound **2**: $(TBA)_4[SiW_{11}O_{39}\{O(Si(CH_2)_3NHCOOC_6H_4NNC_6H_4NO_2)_2\}]$

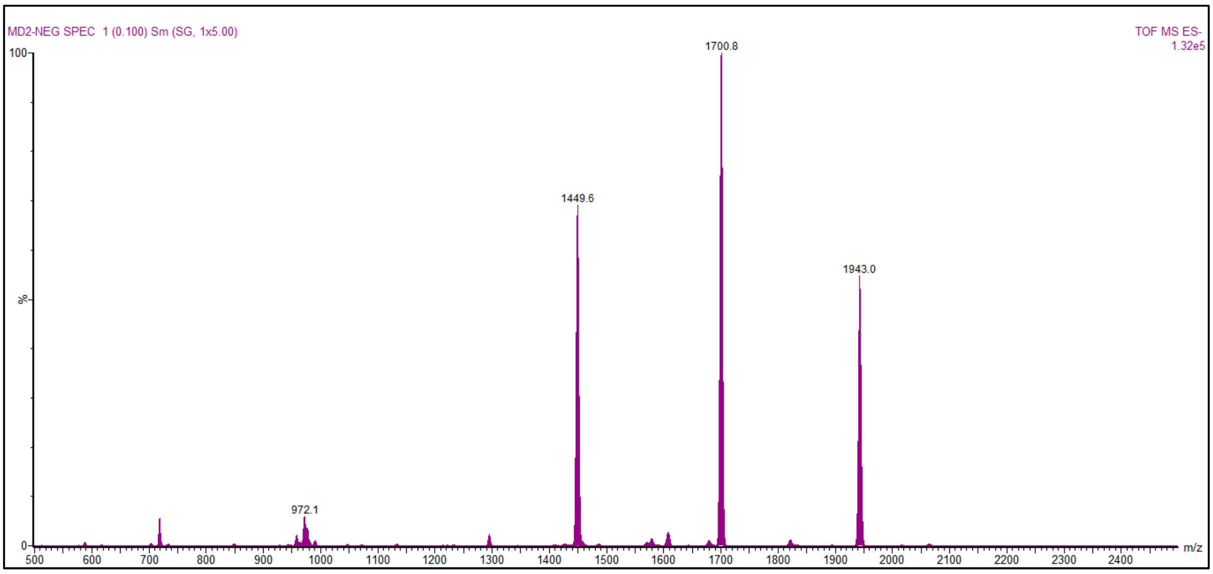

Figure S2. ESI-MS spectrum of compound **2**.

Notable Peaks:

m/z	*z*	*Assignment*
972.1	3-	$\{H[SiW_{11}O_{39}\{O(Si(CH_2)_3NHCO)_2\}]\}$
1449.6	2-	$\{KH[SiW_{11}O_{39}\{O(Si(CH_2)_3NH_2)_2\}]\}$
1700.8	2-	$\{H_2[SiW_{11}O_{39}\{O(Si(CH_2)_3NHCOOC_6H_4NNC_6H_4NO_2)_2\}]\}$
1943.0	2-	$\{(TBA)_2[SiW_{11}O_{39}\{O(Si(CH_2)_3NHCOOC_6H_4NNC_6H_4NO_2)_2\}]\}$

Compound **3**: $(TBA)_4[SiW_{11}O_{39}\{O(Si(CH_2)_3NHCO(CH_2)_5OC_6H_4NNC_6H_5)_2\}]$

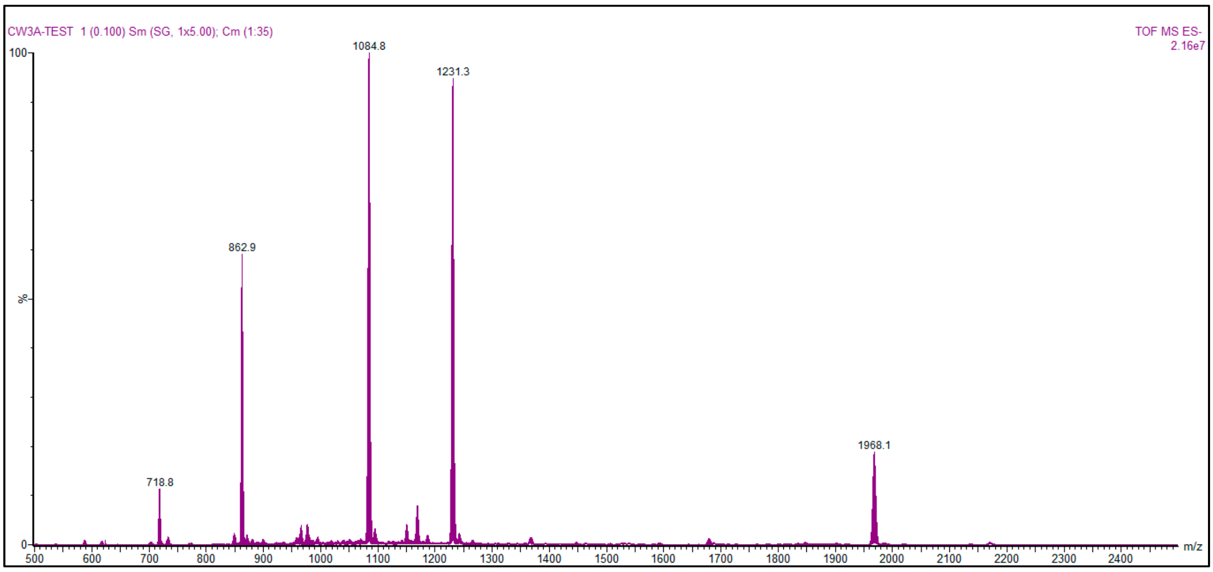

Figure S3. ESI-MS spectrum of compound **3**.

Notable Peaks:

m/z	*z*	*Assignment*
718.8	4-	$[SiW_{11}O_{39}\{O(Si(CH_2)_3NHCO)(SiCH_2CH_2CH_3)\}]$
862.9	4-	$[SiW_{11}O_{39}\{O(Si(CH_2)_3NHCO(CH_2)_5OC_6H_4NNC_6H_5)_2\}]$
1084.8	3-	$\{K[SiW_{11}O_{39}\{O(Si(CH_2)_3NHCO(CH_2)_5OC_6H_4NNC_6H_5)(Si(CH_2)_3NH_2HCl)H_2O\}]\}$
1231.3	3-	$\{(TBA)[SiW_{11}O_{39}\{O(Si(CH_2)_3NHCO(CH_2)_5OC_6H_4NNC_6H_5)_2\}]\}$
1968.1	2-	$\{(TBA)_2[SiW_{11}O_{39}\{O(Si(CH_2)_3NHCO(CH_2)_5OC_6H_4NNC_6H_5)_2\}]\}$

Compound **4**: $(TBA)_4[SiW_{11}O_{39}\{O(Si(CH_2)_3NHCO(CH_2)_{10}OC_6H_4NNC_6H_5)_2\}]$

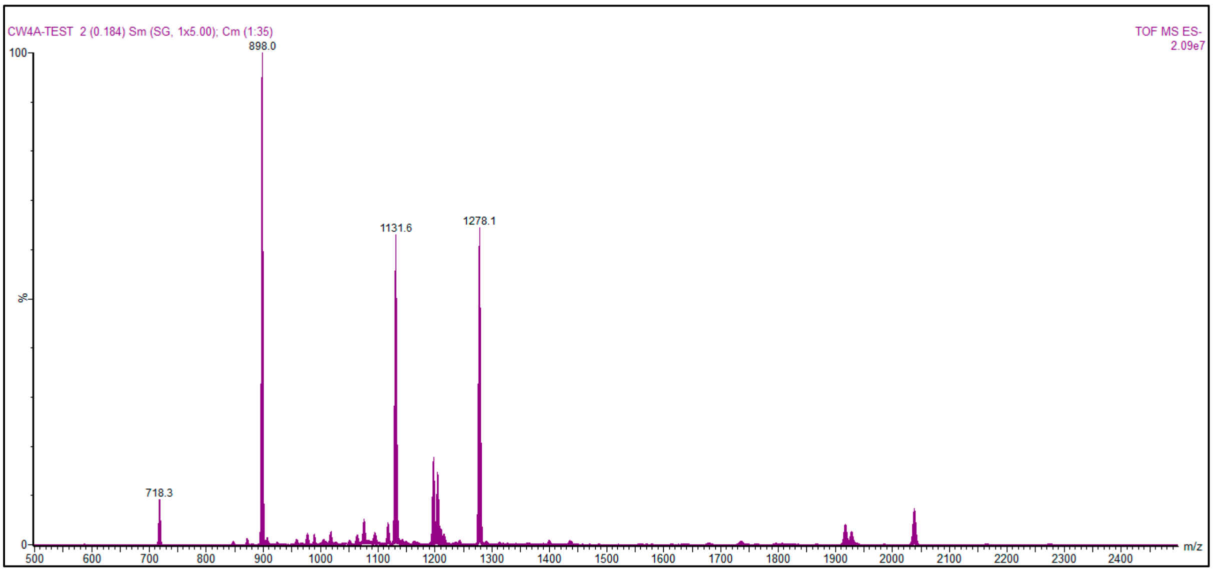

Figure S4. ESI-MS spectrum of compound **4**.

Notable Peaks:

m/z	*z*	*Assignment*
718.3	4-	$[SiW_{11}O_{39}\{O(Si(CH_2)_3NHCO)(SiCH_2CH_2CH_3)\}]$
898.0	4-	$[SiW_{11}O_{39}\{O(Si(CH_2)_3NHCO(CH_2)_5OC_6H_4NNC_6H_5)_2\}]$
1131.6	3-	$\{(TBA)[SiW_{11}O_{39}\{O(Si(CH_2)_3NHCO(CH_2)_{10}OC_6H_4NNC_6H_5)(Si(CH_2)_3NH_2)\}]\}$
1278.1	3-	$\{(TBA)[SiW_{11}O_{39}\{O(Si(CH_2)_3NHCO(CH_2)_{10}OC_6H_4NNC_6H_5)_2\}]\}$
2038.2	2-	$\{(TBA)_2[SiW_{11}O_{39}\{O(Si(CH_2)_3NHCO(CH_2)_{10}OC_6H_4NNC_6H_5)_2\}]\}$

2. 2D IMS/MS summary

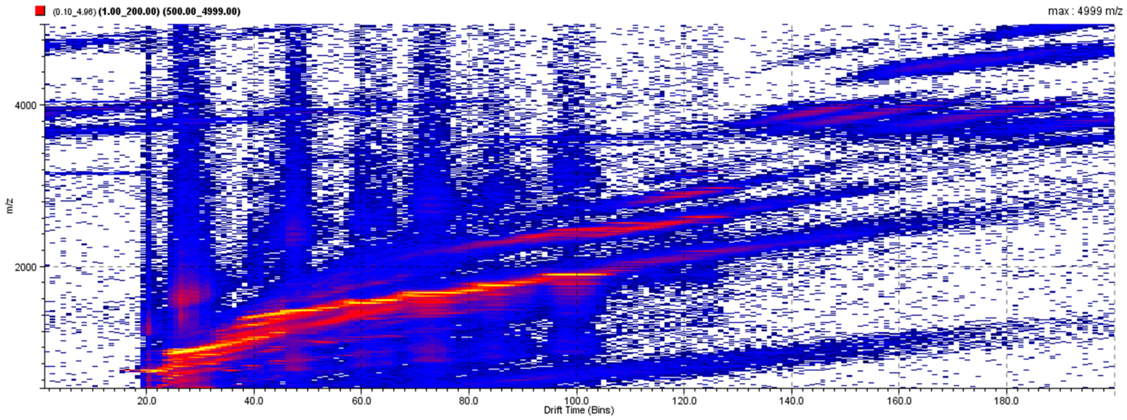

Figure S5. 2D IMS/MS spectrum of compound **1** before UV-irradiation.

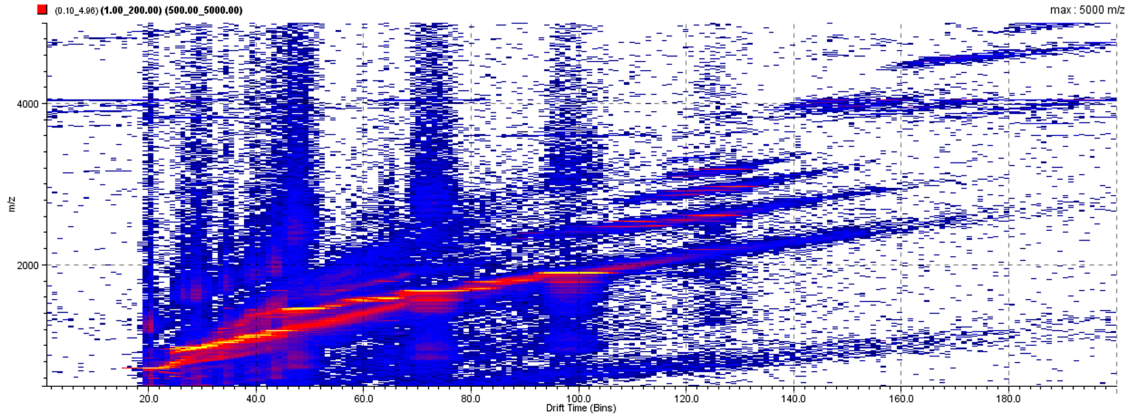

Figure S6. 2D IMS/MS spectrum of compound **1** after UV-irradiation.

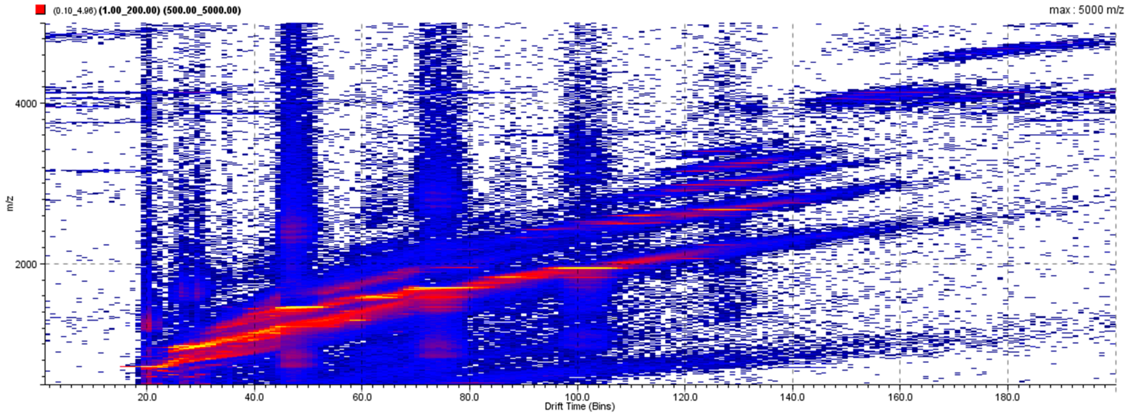

Figure S7. 2D IMS/MS spectrum of compound **2** before UV-irradiation.

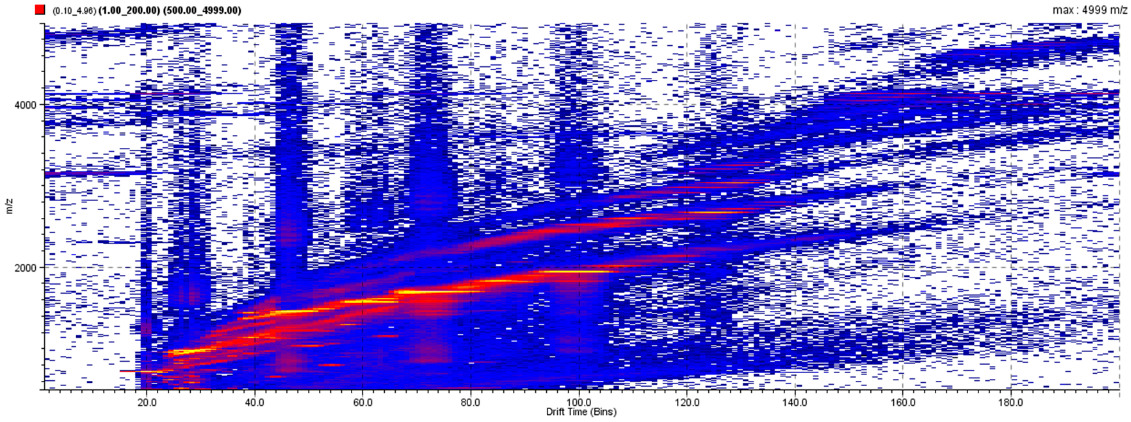

Figure S8. 2D IMS/MS spectrum of compound **2** after UV-irradiation.

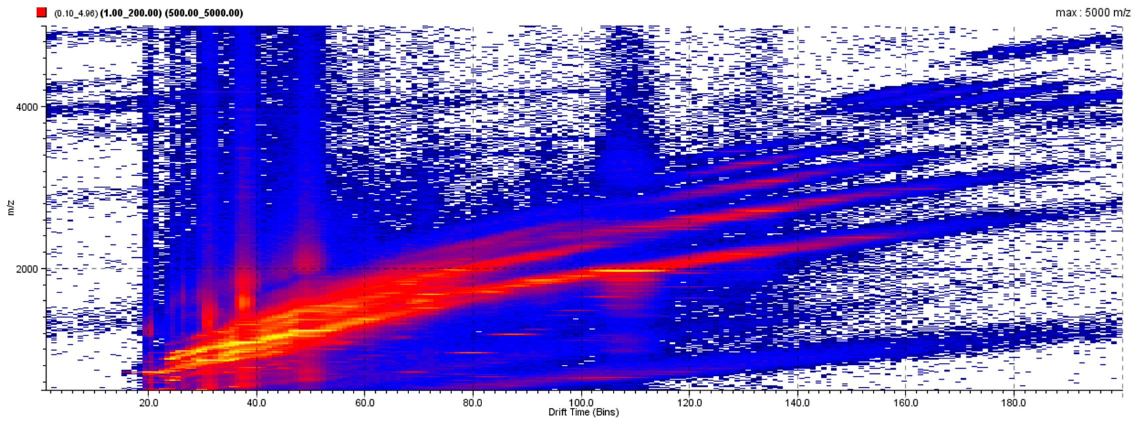

Figure S9. 2D IMS/MS spectrum of compound **3** before UV-irradiation.

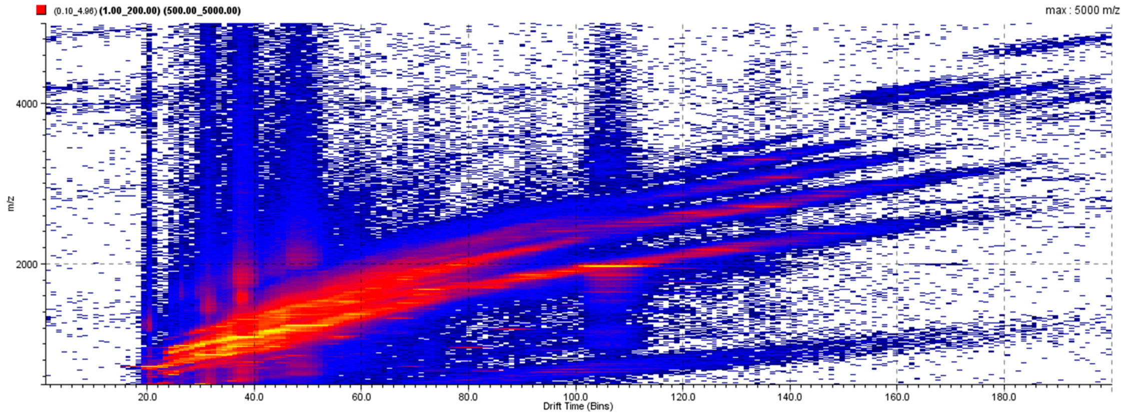

Figure S10. 2D IMS/MS spectrum of compound **3** after UV-irradiation.

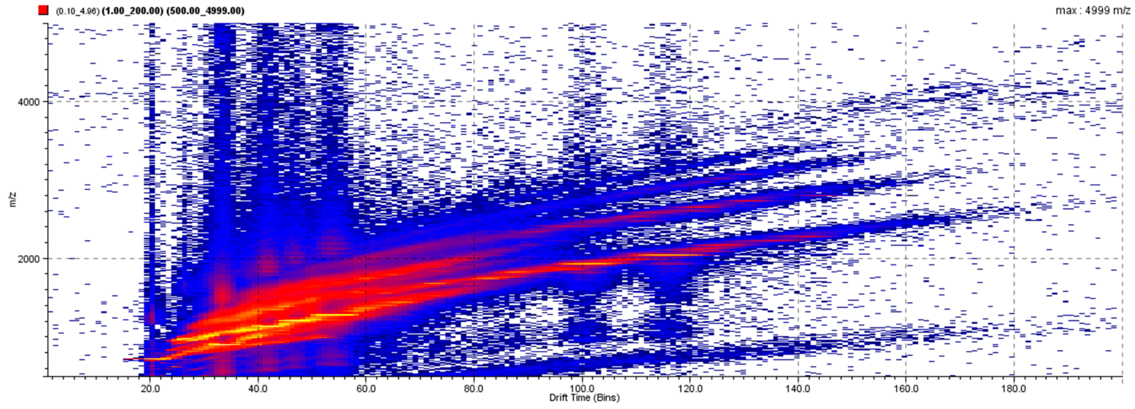

Figure S11. 2D IMS/MS spectrum of compound **4** before UV-irradiation.

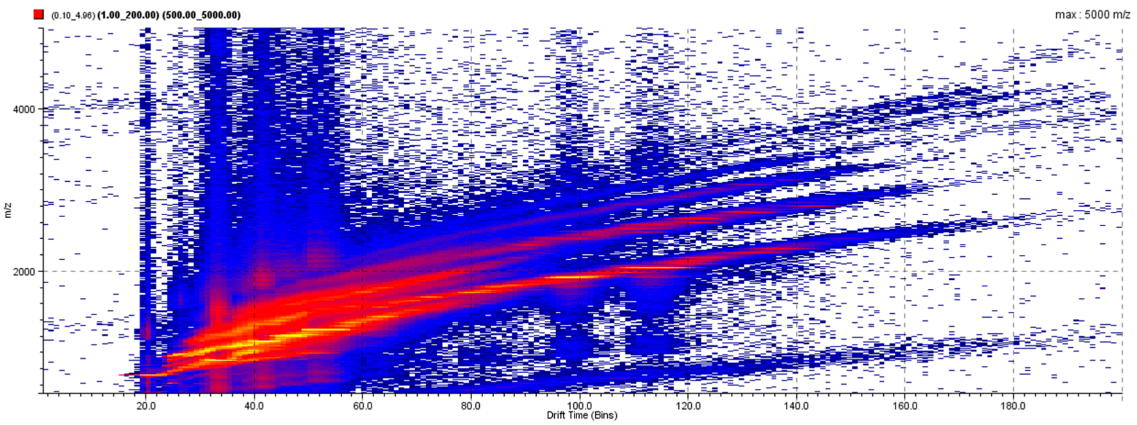

Figure S12. 2D IMS/MS spectrum of compound **4** after UV-irradiation.

3. Drift times and collision cross-sections before and after UV-irradiation

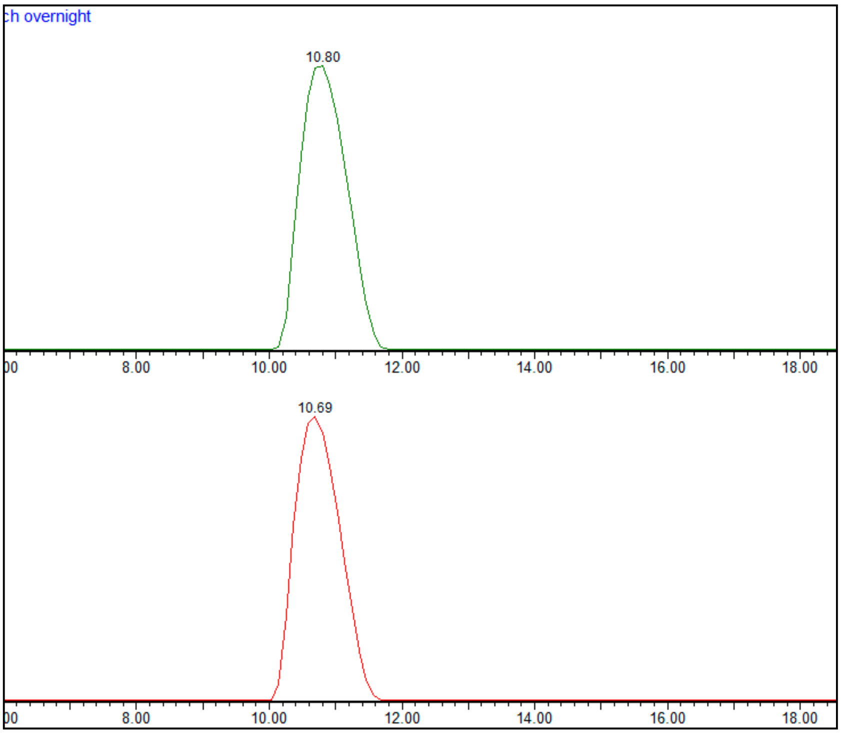

Figure S13. Drift time of compound [**1**+2TBA]$^{2-}$ before and after UV-irradiation.

Table S1. Change in drift time and CCS of monomeric compound [**1**+2TBA]$^{2-}$ when switching between the *trans* and the *cis* conformation of the azo bond.

Isomer	*Drift time (ms)*	*Collision Cross Section (Å^2)*	
trans	10.80	377.48	
cis	10.69	375.04	**ΔCCS = 2.44 Å^2**
trans	10.80	377.48	
cis	10.69	375.04	

trans	10.80	377.48
cis	10.69	375.04

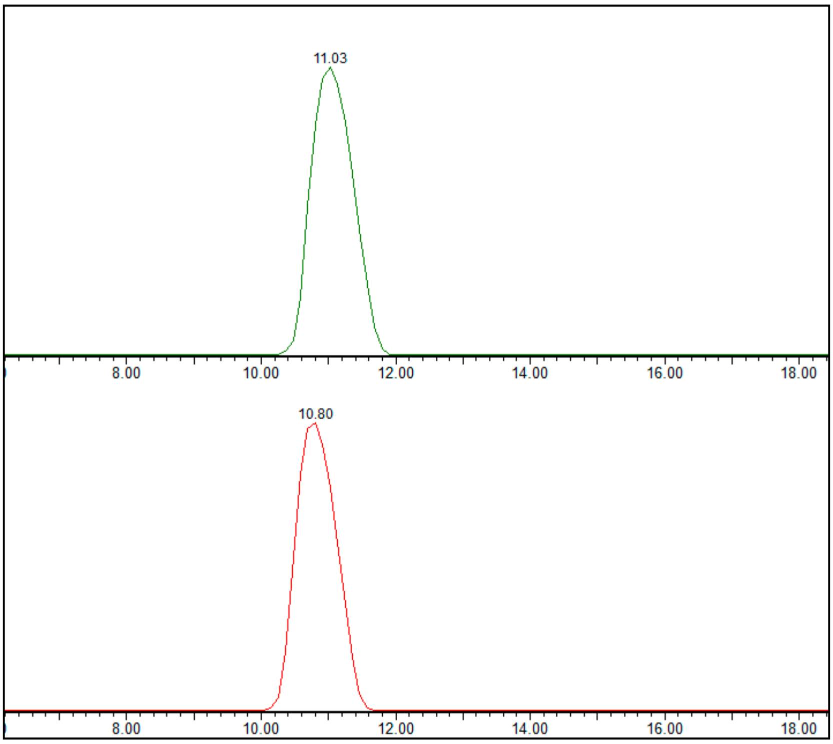

Figure S14. Drift time of compound [**2**+2TBA]$^{2-}$ before and after UV-irradiation.

Table S2. Change in drift time and CCS of monomeric compound [**2**+2TBA]$^{2-}$ when switching between the *trans* and the *cis* conformation of the azo bond.

Isomer	*Drift time (ms)*	*Collision Cross Section (Å^2)*
trans	11.03	382.52
cis	10.80	377.45
trans	11.03	382.52
cis	10.80	377.45
trans	11.03	382.52
cis	10.80	377.45

ΔCCS = 5.07 Å^2

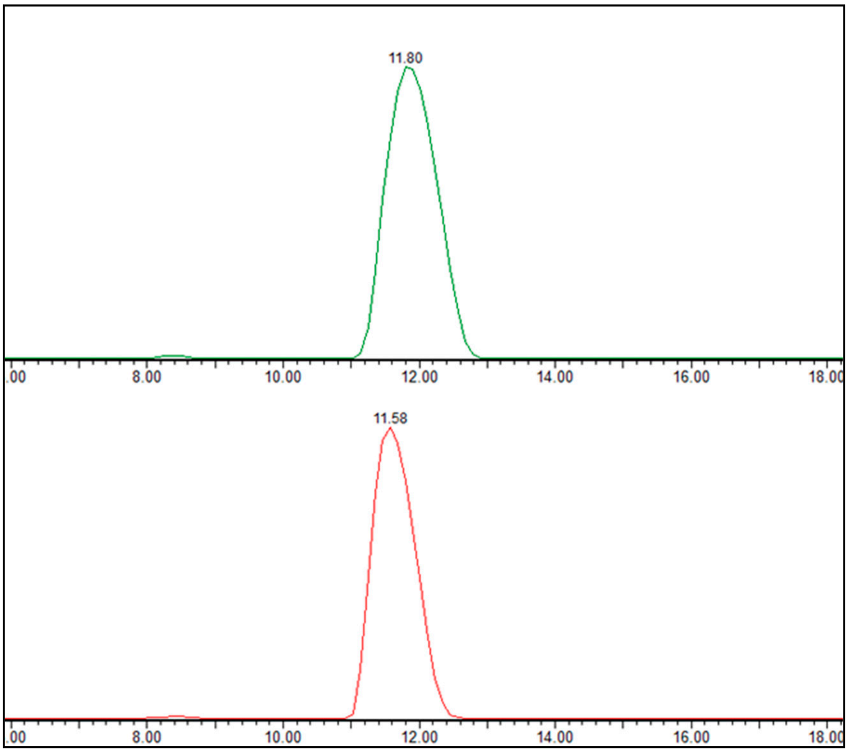

Figure S15. Drift time of compound [**3**+2TBA]$^{2-}$ before and after UV-irradiation.

Table S3. Change in drift time and CCS of monomeric compound [**3**+2TBA]$^{2-}$ when switching between the *trans* and the *cis* conformation of the azo bond.

Isomer	*Drift time (ms)*	*Collision Cross Section (Å^2)*
trans	11.80	399.19
cis	11.58	394.46
trans	11.80	399.19
cis	11.58	394.46
trans	11.80	399.19
cis	11.58	394.46

ΔCCS = 4.73 Å^2

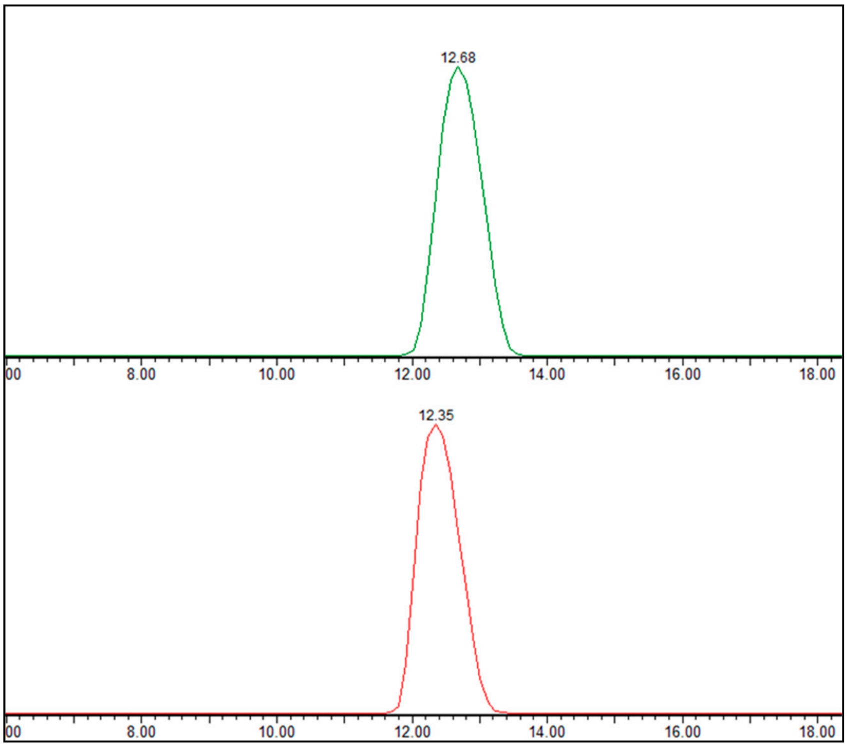

Figure S16. Drift time of compound [**4**+2TBA]$^{2-}$ before and after UV-irradiation.

Table S4. Change in drift time and CCS of monomeric compound [**4**+2TBA]$^{2-}$ when switching between the *trans* and the *cis* conformation of the azo bond.

Isomer	*Drift time (ms)*	*Collision Cross Section (Å^2)*	
trans	12.68	417.73	
cis	12.35	410.82	
trans	12.68	417.73	**ΔCCS = 6.91 Å^2**
cis	12.35	410.82	
trans	12.68	417.73	
cis	12.35	410.82	

4. Synthesis and Characterization of compounds 1 and 2

4.1 Synthesis of 4-(phenyldiazenyl)phenol.

At room temperature, 3.7 g aniline (40 mmol) was dissolved in 100 ml deionized water. Added 10 ml of hydrochloric acid to it and magnetic stirred. After the resulting mixture was clarified, put it in an ice bath and kept the temperature below 5°C. Then added 3.8 g sodium nitrite solution dissolved in 10 ml deionized water, kept it in ice bath, and stirred for 30 minutes,3.7 g phenol solution dissolved with a mixed solution of 10 ml CH_3OH and 20 ml H_2O was added dropwise followed by stirring. The pH was then adjusted to 8 by adding

NaOH aqueous solution (1M), removed ice bath and stirred at room temperature for 2 hours. The yellow precipitate was collected by filtration, washed thoroughly with deionized water and vacuum dried, the yellow solid product was about 7.4 g. ^{1}H NMR ($CDCl_3$-d_3, 400 MHz), δ (ppm): 8.10 (d, 2H), 7.83 (d, 2H), 7.65 (s, 2H), 7.26 (s, 1H), 7.02 (d, 2H), 5.34 (s, 1H).

4.2 Synthesis of 4-((4-nitrophenyl) diazinyl) phenol

At room temperature, 5.2 g para-nitroaniline (40 mmol) was dissolved in 100 ml deionized water. Added 10 ml of hydrochloric acid to it and magnetic stirred. After the resulting mixture was clarified, put it in an ice bath and kept the temperature below 5°C. Then added 3.8 g sodium nitrite solution dissolved in 10 ml deionized water, kept it in ice bath, and stirred for 30 minutes,3.7 g phenol solution dissolved with a mixed solution of 10 ml CH_3OH and 20 ml H_2O was added dropwise followed by stirring. The pH was then adjusted to 8 by adding NaOH aqueous solution (1M), removed ice bath and stirred at room temperature for 2 hours. The yellow precipitate was collected by filtration, washed thoroughly with deionized water and vacuum dried, the yellow solid product was about 6.8 g. ^{1}H NMR ($CDCl_3$-d_3, 400 MHz), δ (ppm): 8.30 (d, 2H), 7.85-7.81 (dd, 4H), 7.03 (d, 2H), 5.35 (s, 1H).

4.3 Synthesis of $(OEt)_3SiCH_2CH_2CH_2NHCOOC_6H_4N{=}NC_6H_5)_2$

(1) To a mixture of 4-(phenyldiazenyl)phenol (0.5 g) and IESPIC solution in 50 mL THF was added trientylamine (0.7 mL) under argon atmosphere in a 100 mL three-necked flask, heated and stirred, and then refluxed for 24 hours. Stopped heating when TLC detected only one point and concentrated under reduced pressure. Vacuum dried afforded product for reserving in 78.6% yields (0.88 g). IR (KBr, cm^{-1}): ν =3289 (m), 2973 (m), 2881 (m), 1697 (vs), 1531 (s), 1494 (s), 1251 (m), 1224 (s), 1104 (s), 1080 (s), 956 (w), 768 (w). ^{1}H NMR (DMSO-d_6, 400 MHz), δ (ppm): 7.91 (d, ArH, 4H), 7.59 (d, ArH, 3H), 7.32 (s, ArH, 2H), 3.76 (q, CH_2, 6H), 3.06 (q, CH_2, 2H), 1.55 (s, CH_2, 2H), 1.16 (t, CH_3, 9H), 0.58 (t, CH_2, 2H).

(2) The synthesis of $(OEt)_3SiCH_2CH_2CH_2NHCOOC_6H_4N{=}NC_6H_4NO_2)_2$ was similar as above except by using 4-((4-nitrophenyl) diazinyl) phenol instead of 4- (phenyldiazenyl)phenol. IR (KBr, cm^{-1}): ν =3321 (m), 2973 (m), 2927 (m), 2882 (m), 1705 (vs), 1612 (w), 1542 (s), 1493 (s), 1346 (vs), 1280 (w), 1256 (w), 1222 (vs), 1103 (s), 1077 (s), 953 (m), 863 (m), 754 (w), 683 (w). ^{1}H NMR (DMSO-d_6, 400 MHz), δ (ppm): 8.44 (d, ArH, 2H), 8.08 (d, ArH, 2H), 8.01 (d, ArH, 2H), 7.96 (s, NH, 1H), 7.38 (d, ArH, 2H), 3.76 (q, CH_2, 6H), 3.08 (q, CH_2, 2H), 1.56 (s, CH_2, 2H), 1.16 (t, CH_3, 9H), 0.60 (t, CH_2, 2H).

4.4 Characterization of compounds **1** and **2**

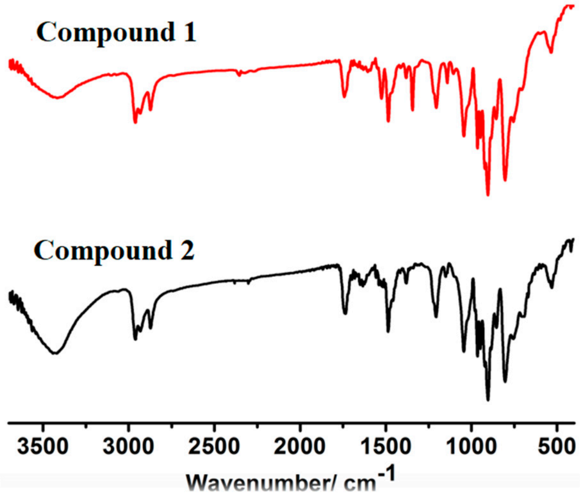

Figure S17. FT-IR spectra of compounds **1** and **2**

For compound 1: IR (KBr, cm^{-1}): ν = 3442 (m), 2961 (m), 2872 (m), 1736 (m), 1486 (m), 1206 (m), 1044 (s), 1044 (m), 964 (s), 947 (s), 903 (vs), 854 (m), 803 (s).

For compound 2: IR (KBr, cm^{-1}): ν = 3418 (m), 2961 (m), 2873 (m), 1742 (m), 1525 (m), 1485 (m), 1344 (m), 1205 (m), 1141 (w), 1044 (m), 964 (s), 947 (s), 904 (vs), 856 (m), 804 (s), 535 (w).

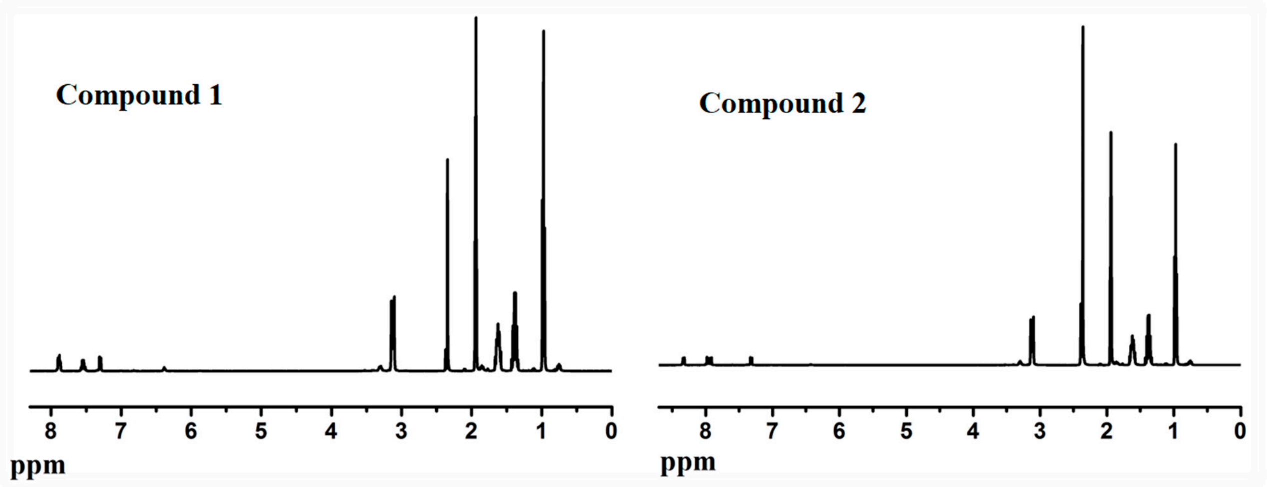

Figure S18. ^{1}H NMR spectra of compounds **1** and **2**

For compound 1: ^{1}H NMR (CD_3CN-d_3, 400 MHz, ppm) δ = 7.88 (d, ArH, 8H), 7.54 (d, ArH, 6H), 7.29 (s, ArH, 4H), 6.38 (t, NH, 2H), 3.29 (t, CH_2, 4H), 3.12 (t, CH_2, 32H), 1.85 (s, CH_2, 4H), 1.62 (q, CH_2, 32H), 1.38 (t, CH_2, 32H), 0.97 (t, CH_3, 48H), 0.75 (t, CH_2, 4H).

For compound 2: ^{1}H NMR (CD_3CN-d_3, 400 MHz, ppm) δ = 8.32(d, ArH, 4H), 7.97 (d, ArH, 4H), 7.97 (d, ArH, 4H), 7.92 (d, ArH, 4H), 7.31 (d, ArH, 4H), 3.29 (t, CH_2, 4H), 3.12 (t, CH_2, 32H), 1.86 (s, CH_2, 4H), 1.62 (q, CH_2, 32H), 1.37 (m, CH_2, 32H), 0.97 (t, CH_3, 48H), 0.76 (t, CH_2, 4H).

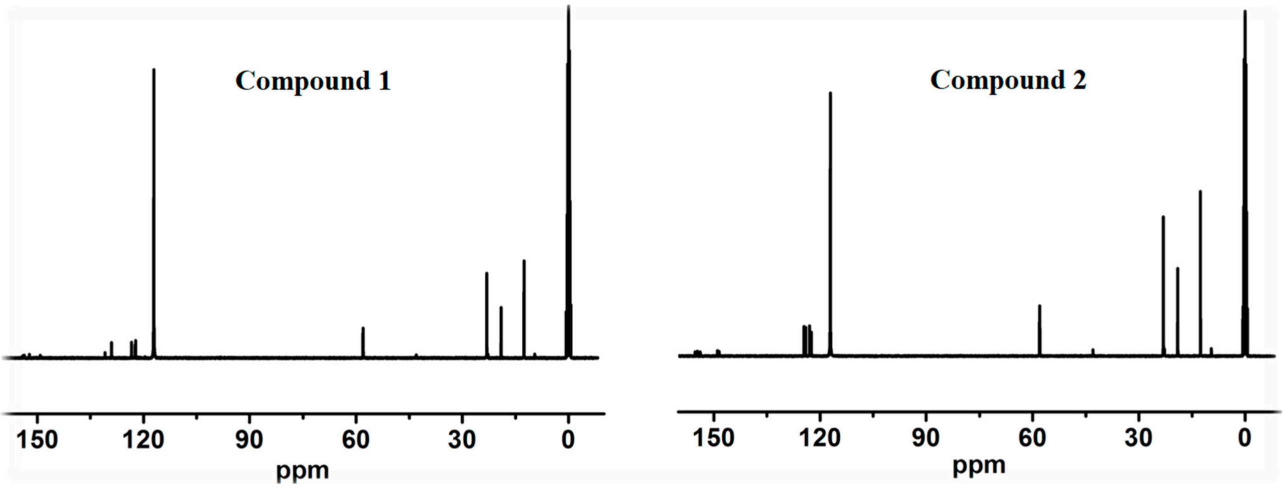

Figure S19. ^{13}C NMR spectra of compounds **1** and **2**

For compound 1: ^{13}C NMR (CD_3CN-d_3, 100 MHz, ppm) δ = 155.1, 154.8, 153.3, 150.3, 132.0, 130.1, 124.5, 123.4, 123.3, 59.1, 44.2, 24.2, 23.9, 20.2, 13.7, 10.6.

For compound 2: ^{13}C NMR (CD_3CN-d_3, 100 MHz, ppm) δ = 156.6, 155.8, 155.0, 150.2, 149.7, 125.8, 125.3, 124.2, 123.7, 59.2, 44.1, 24.3, 24.0, 20.3, 13.9, 10.8.

5. Photo-responsive conformational variation behaviors of compound 2

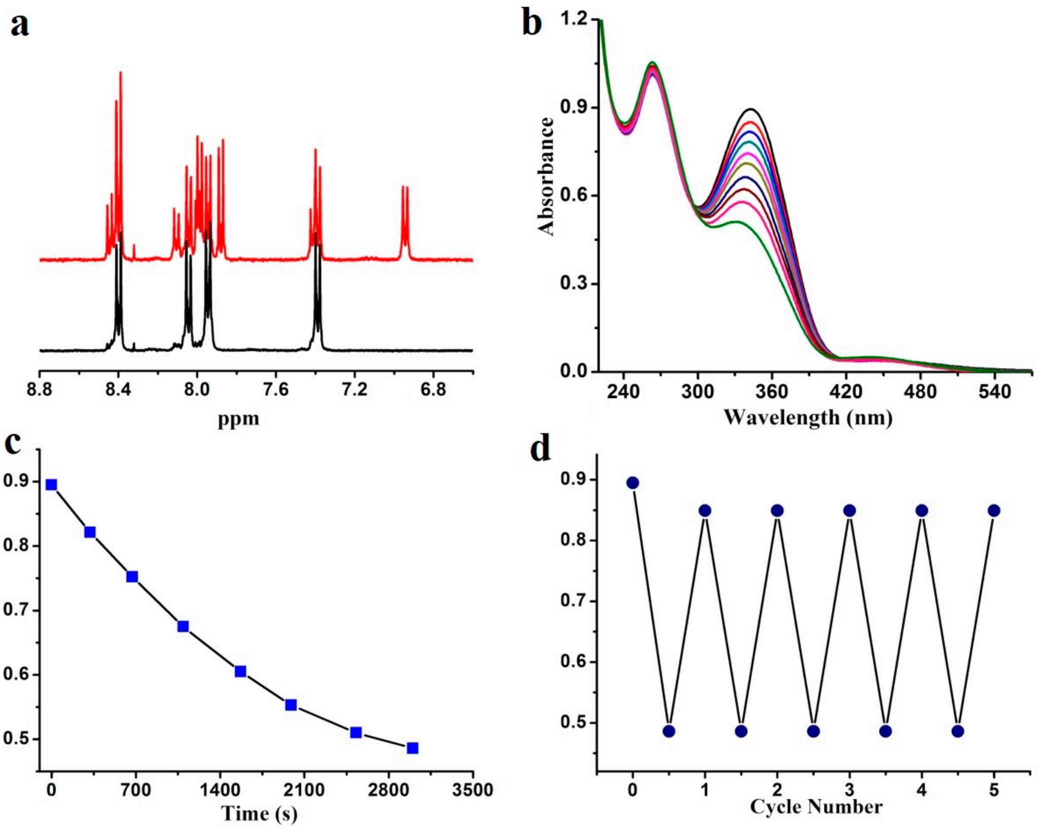

Figure S20. (a) ^{1}H NMR spectra of compound **2** in d_3-CH_3CN before (bottom) and after (top) UV irradiation at 365 nm. (b) UV/Vis spectra of compound **2** in CH_3CN. (c) The kinetic curve of the UV absorbance with time

increasing. (d) The UV absorbance at 343 nm upon alternating UV irradiation at 365 nm and visible light for five cycles.

Note: The conformational variation behaviors of other compounds (1, 3, 4) were reported in the *ChemPlusChem* **2013,** *78,* 1226.

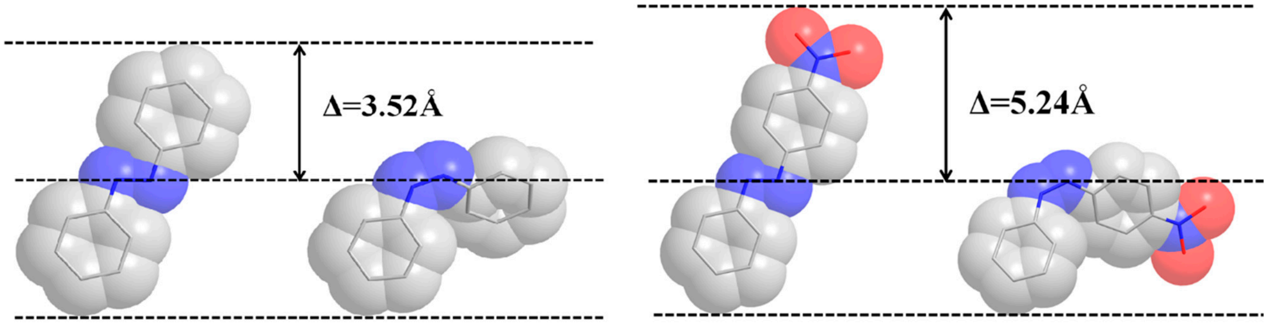

Figure S21. The models of azo and nitro-azo groups with *trans*- and *cis*- conformations. The models were constructed by ChemBio3D Software and the shape difference was roughly calculated by atom distance.
